# Increasing Awareness and Engagement in Research Among Psychiatry Trainees Using a Quality Improvement (QI) Approach

**DOI:** 10.1192/bjo.2026.11385

**Published:** 2026-06-30

**Authors:** Ebraam Gerges, Ruth Micheal Elaigwu, Deepa Krishnan

**Affiliations:** Nottinghamshire NHS Foundation Trust, Nottingham, United Kingdom

## Abstract

**Aims:**

To improve awareness, confidence, and engagement in research among psychiatry trainees. Our SMART aim was that by August 2026, at least 75% of trainees would report moderate or higher confidence in research on post-intervention Likert scale questionnaires.

**Methods:**

Baseline data were collected using questionnaires distributed to Core (CT1–CT3) and Higher (ST4–ST6) trainees. Measures included awareness of research contacts, understanding of ARCP requirements, current involvement in research, and self-rated confidence. A fishbone analysis identified key barriers across people, process, workload, communication, training, and organisational domains. Stakeholders included Training Programme Directors, research-active consultants, Trust R&D teams, and academic partners. Interventions were designed using QI methodology and driver diagrams.

**Results:**

Interest in research was high across all trainees. However, core trainees demonstrated low awareness of research contacts, processes, and ARCP requirements, with limited active involvement. Higher trainees reported greater awareness and participation but continued to identify barriers including lack of time, mentorship, and clear guidance. Confidence scores were low–moderate in core trainees and moderate in higher trainees.

**Conclusion:**

There is a clear gap between trainee interest in research and actual engagement. System-level interventions focusing on awareness, mentorship, and access to opportunities are expected to improve research confidence and participation. This project aims to foster a sustainable research culture within psychiatry training and support trainees in meeting curriculum and ARCP requirements.

